# Molecular detection of small hive beetle *Aethina tumida* Murray (Coleoptera: Nitidulidae): DNA barcoding and development of a real-time PCR assay

**DOI:** 10.1038/s41598-018-27603-x

**Published:** 2018-06-25

**Authors:** Dongmei Li, David W. Waite, Qing-Hai Fan, Sherly George, Linda Semeraro, Mark J. Blacket

**Affiliations:** 1 Plant Health and Environment Laboratory, Diagnostic and Surveillance Services, Ministry for Primary Industries, P.O. Box 2095, Auckland, 1140 New Zealand; 2 Agriculture Victoria, AgriBio, Centre for AgriBioscience, Bundoora, Victoria, 3083 Australia; 3 Present Address: School of Biological Sciences, University of Auckland, Auckland, New Zealand

## Abstract

Small hive beetle (SHB), *Aethina tumida* can feed on honey, pollen and brood in honey bee colonies. It was endemic to Africa, but since 1996 has been detected in a number of countries worldwide, including Australia, Brazil, Canada, Italy, Mexico, South Korea, Philippines and the USA where it has had economic effects on local apiculture. To improve SHB identification, we obtained the first reference sequences from the DNA barcoding 5′ COI gene region for SHB and some species of the family Nitidulidae associated with beehives. Phylogenetic analysis of SHB COI sequences (3′ COI) revealed two divergent lineages, with those from Australia and USA being genetically different from the recent detection in Italy. Many countries, including New Zealand, are currently free from SHB, and require a rapid detection method for biosecurity. Here we present the development and validation of a real-time PCR assay for detection of SHB. The assay showed high specificity and sensitivity for detecting SHB, with no cross-reaction observed with closely related species, such as *A*. *concolor*. The real-time PCR is sensitive, detecting the target sequences up to 100 copies/µL. This assay should prove a useful biosecurity tool for rapid detection of SHB worldwide.

## Introduction

The family Nitidulidae comprises roughly 4,000 species worldwide^1^. A number of the nitidulid beetles are associated with bee hives (Table 1), however, few impacts on honey bee health have been reported^1–5^, and benign associations between several *Brachypeplus* species and honey bees have been reported. *Brachypeplus auritus* Murray, lives as larva and adult in Australian wild bee hives and feeds on honey and wax^6^ and *B*. *macleayi* Murray is common in hives feeding on spilt pollen^7^. Recently, *B*. *basalis* Erichson, of Australian-origin^8^, has been shown to infest honeybee hives in the USA, with damage limited to date^9,10^. Most other associations of nitidulid beetles within beehives appear to be non-detrimental (Table 1). Another species, however, small hive beetle (SHB) *Aethina tumida* (Murray, 1867) (Coleoptera: Nitidulidae), is considered a serious pest of beehives^11,12^.

**Table 1:** Species of Nitidulidae (and Cryptophagidae) beetles previously associated with beehives worldwide.

Species (Family)	Reported Pest Status	BOLD barcode* (public)	Present in Australia	Present in NZ
*Aethina tumida* Murray (Nitidulidae)	Breeds in hives, damaging beehive pest^16,18^	5 (5)	Yes, Cosmopolitan	No, Exotic
Aethina concolor** (Macleay) (Nitidulidae)	Beehive/hibiscus flowers^28,65,66^	2 (2)	Yes, Endemic	Yes, Adventive
*Brachypeplus* sp.*** (Nitidulidae)	Breeds in hives, potential beehive pest^9,67^	Genus: 5 (4)	Yes, Cosmopolitan	Yes
*Carpophilus dimidiatus* (Fabricius) (Nitidulidae)	Shelter/food^68,69^	3 (1)	Yes, Cosmopolitan	Yes, Adventive
*Carpophilus hemipterus* (Linnaeus) (Nitidulidae)	Accidental^70–72^	33 (9)	Yes, Cosmopolitan	Yes, Adventive
*Carpophilus lugubris* Murray (Nitidulidae)	Potential beehive pest^4,72^	4 (2)	No, Exotic. (Genus present)	No, Exotic
*Cychramus luteus* (Fabricius) (Nitidulidae)	Shelter/food^5^	28 (21)	No, Exotic. (Genus present)	No, Exotic
*Epuraea corticina* Erichson (Nitidulidae)	Shelter/food^69^	7 (2)	No, Exotic. (Genus present)	No, Exotic
*Epuraea (Haptoncus) luteola* (Erichson) (Nitidulidae)	Potential beehive pest^73^	1 (0)	No, Exotic. (Species occurs on Christmas Island)	No, Exotic
*Glischrochilus fasciatus* Olivier (Nitidulidae)	Shelter/food^69^	10 (10)	No, Exotic	No, Exotic
*Lobiopa insularis* (Laporte) (Nitidulidae)	Shelter/food^69–71^	Genus: 2 (2)	No, Exotic	No, Exotic
*Urophorus humeralis* (Fabricius) (Nitidulidae)	Accidental^70,71,74^	3 (1)	Yes, Cosmopolitan	Yes, Cosmopolitan
*Cryptophagus hexagonalis* Tournier (Cryptophagidae)	Breeds in hives, benign^53^	Genus: 213 (184)	No, Exotic. (Genus present)	No, Exotic

Note:

^*^BOLD database accessed 19 April 2017.

^**^One of the *Aethina concolor* specimen (T16_01444 in Table 2) was obtained from beehive via New Zealand general surveillance program. Another *A*. *concolor* specimen (T17_00549 in Table 2) was collected from and *Calystegia sepium* flowers. The survey of Buchholz *et al*.^65^ showed that *A*. *concolor* individuals were observed on blossoms which were regularly visited by honey bees.

^***^Brachypeplus brevicornis is present in New Zealand^67^.

The small hive beetle is native to sub-Saharan Africa, where it was originally described as a minor pest of the western honey bees^13^. However, SHB was detected in the USA in 1996^14^ and Australia (excluding Tasmania) in 2002 where it is now established as an invasive pest^12,15–18^. SHB were also found in Canada in 2002, Mexico in 2007 and Hawaii in 2010^16,19,20^. Recently, SHB was introduced to Italy where it has already caused significant damage to apicultural industries^1,11^, and raised concern that SHB populations might be able to establish in Europe^1,11^. The recent detections of SHB in Philippines^21^, South Korea^22^ and Brazil^23^ indicate that SHB has the potential to become a global issue for apiculture and wild bee populations.

The SHB is a nest parasite and scavenger of the honeybee, *Apis mellifera* Linnaeus^24,25^. Of around 30 species of *Aethina* known to occur worldwide^25,26^, 14 of which occur in Australia^27,28^, only the SHB and *A*. *concolor* Macleay are known to be associated with beehives. The SHB invades beehives, where it lays eggs in the capped brood cells, or in the small cracks or crevices around the hive; weakened, stressed or queenless hives being particularly susceptible^29^. The larvae are the most damaging stage and tunnel through comb with stored honey or pollen, damaging or destroying cappings and combs. Larvae defecate in honey and the honey becomes discoloured from the faeces. In severe infestations, honey will ferment and leak from cells^12,17^. Bees often desert affected hives at this point and beekeeping equipment may be unsalvageable^30^. SHB is also attracted to stored honey^12,17^. The queen bee industry in the USA has lost export markets due to SHB and quarantine restrictions in some countries^31,32^. SHB can mass reproduce in honey bee colonies and in Italy they are known to have led to the collapse of the entire bee nest in less than five days^33,34^. Further reports also suggest that SHB could invade other bee species colonies, such as *Austroplebeia australis* Friese^35^ and *Bombus impatiens* Cresson^2,36^.

Honey bees are vitally important for the pollination of agricultural crops; the results of decreased honey bee populations will cause the decreasing of crop yields and fruit quality^32,37^. Varroa mite, *Varroa destructor* Anderson and Trueman have been established in New Zealand since 2000^38^, while there is no evidence that Varroa has established in Australia. With the *V*. *destructor* infestation, the deformed wing virus (DWV) may become the major virus causing the collapse of the honey bee colonies^39–41^. Thus, the combined effects of SHB and *V*. *destructor* would potentially reduce the populations of both feral and managed honey bee colonies as they appear to have in the United States^32^. In addition, DWV was also recently detected in adult SHB, indicating that SHB is a potential biological vector of honeybee viruses^42^. Thus, if SHB were to establish in New Zealand it could cause serious damage to the local honeybee industry.

The SHB was first detected in Australia in October 2002 in large numbers and wide distribution^15,16,18^. Since then, SHB has become a significant seasonal pest for beekeeping industries throughout the warm and humid coastal strip between Victoria and North Queensland. New Zealand is currently SHB-free, potential introductions into New Zealand are considered a serious threat to the local beekeeping industry^15,17^. New Zealand and Australia share a relatively recent history of *Apis* and *Bombus* introductions (*Bombus* only occurs in New Zealand and Tasmania), with overlap of economically important crops, native plant genera, and biogeographical history^43^. Early detection of exotic pests is important and will help maintain effective biosecurity controls^44^. Therefore it is necessary to develop a rapid detection assay for identification of any intercepted suspect SHB samples.

**Figure 1:**
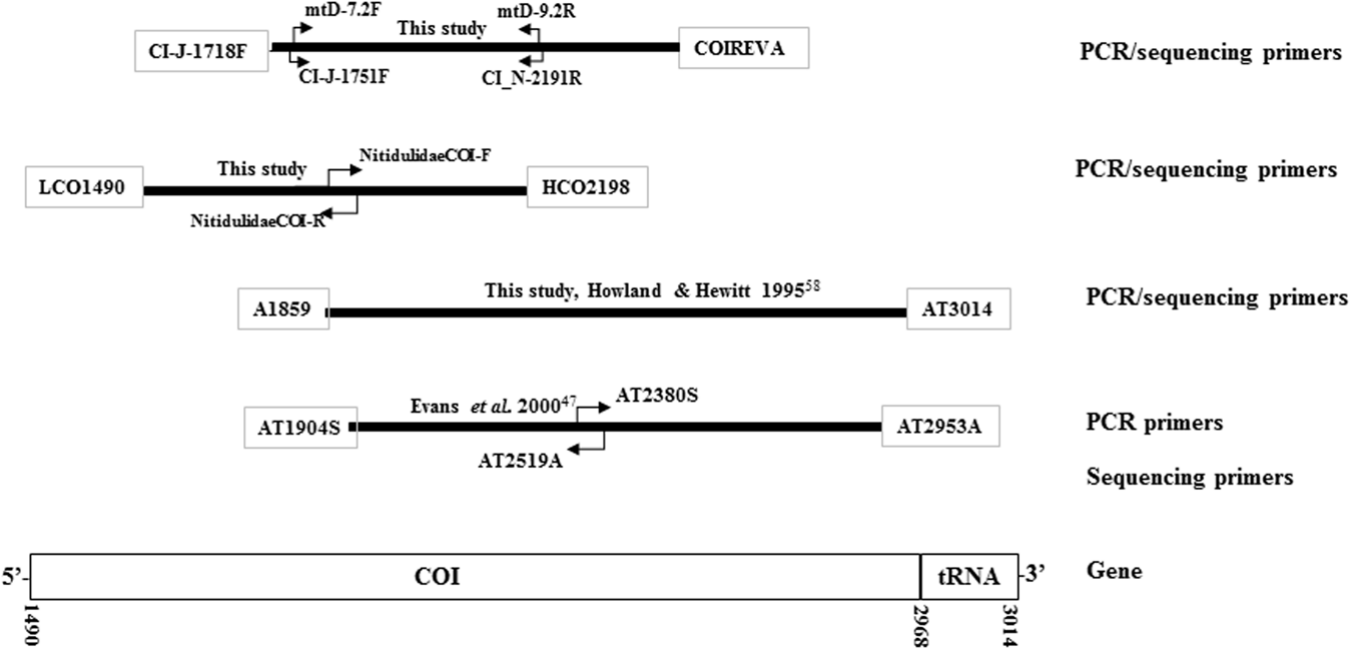
Comparison of primer locations for PCR and sequencing of the COI gene from Mitochondrial DNA studies on SHB. The position number of the primers refer to the positions of the *Drosophila yakuba* mitochondrial genome^64^.

The main aims of the current study were to develop improved methods for molecular detection of SHB. DNA barcoding of insects is commonly used for identification^45^, in which universal primers^46^ are used to amplify the 5′ fragment of mitochondrial Cytochrome Oxidase I (COI) gene region or the “Folmer” region (~700 bp) (Fig. 1). However, no complete Folmer region sequences for SHB have been published to date, SHB COI sequences available are mainly targeting the 3′ region of COI, from the position of 1859 onwards^20,47,48^, with <260 bp overlap within the Folmer region (Fig. 1). Therefore in the current study a DNA barcoding library of the Folmer region for SHB and some related species was established. Ward *et al*.^48^ reported a real-time PCR protocol for rapid detection of SHB^48^, however, mismatches in the primer binding region were observed with additional SHB COI sequences published, therefore there is the need to develop a new real-time PCR assay to be able to amplify all the SHB samples detected so far. Here, we developed a TaqMan real-time PCR assay for rapid detection of SHB and validated it according to the MIQE guidelines^49^. This assay will be of great assistance in the early detection of SHB and provide useful information for the biosecurity decision making.

## Results

### DNA barcoding

DNA barcoding sequences for SHB samples and related species obtained in the bee surveys from Australia and specimens intercepted at New Zealand are listed in Table 2. DNA sequences of 5′fragment of COI or the Folmer region obtained in this study showed over 99% identities between the SHB samples from Australia and USA, but they shared around 94–96% identities with the SHB from Italy. Those SHB sequences were distantly related (>17% differences) to those of *A*. *concolor* (5 sequences: T16_01444, T17_00549, AQ5, GU217509 & GU217510) and *A*. *suturalis* (9 sequences: KJ480785–KJ480793), with around 81–83% identity. The sequences of those SHB is also quite divergent from the other nitudulid beetles collected from bee surveillance survey (Table 2). Of the existing 79 SHB COI sequences (for the 3′ fragments region) deposited in GenBank, however, there was little overlap with the Folmer DNA barcoding region (Fig. 1).

**Table 2:** Species associated with beehives and nitidulid beetles for DNA barcoding and tested in the real-time *Aethina tumida* PCR assay.

Species ID* (Family)	Voucher ID**	n	Origin	Year	Life stage	Origin	Match to SHB, %^***^	PCR results	BOLD/GenBank Accession #
*Aethina tumida* Murray (Nitidulidae)	VAITC5359-5366	9	Australia	2015	adult	Beehive	99–100	+	ANZN012-17 to ANZN018-17
	AQ3	2	Australia	2014	adult	Intercepted		+	ANZN002-17
	AQ4	1	USA	2015	larva	Beehive		+	ANZN001-17
	T17_01583A	5	Italy	2015–2016	adult	Beehive	94–96	+	ANZN026-17 to ANZN029-17
	T17_01583B	5	Italy	2016–2017	larva	Beehive	94–96	+	ANZN030-17 to ANZN033-17
*Aethina concolor* (Macleay) (Nitidulidae)	T16_01444	1	New Zealand	2016	adult	Beehive	81–82	−	ANZN006-17
	T17_00549	1	New Zealand	2017	adult	Collection		−	ANZN008-17
	AQ5	1	New Zealand	2017	adult	Collection		−	ANZN007-17
*Brachypeplus* sp. (Nitidulidae)	VAITC5397–5399	3	Australia	1999/2003	adult	Beehive		−	ANZN021-17 to ANZN023-17
*Brachypeplus* sp. (Nitidulidae)	20/09/1992	1	Hawaii	1992	adult	Intercepted	n/a	−	ns
*Brachypeplus* sp. (Nitidulidae)	Australia 18401	1	Australia	—	larva	Intercepted	n/a	−	ns
	T16_02506	1	Australia	2016	adult	Intercepted	85	−	ANZN003-17
*Carpophilus dimidiatus* (Fabricius) (Nitidulidae)	T11_3082	1	China	2011	adult	Intercepted	n/a	−	ns
*Carpophilus gaveni* Dobson (Nitidulidae)	T11_02455	1	New Zealand	2011	adult	*Pyrus* sp.	n/a	−	ns
*Carpophilus hemipterus* (Linnaeus) (Nitidulidae)	VAITC5395	1	Australia	2003		Beehive		nt	
*Carpophilus mutilatus* Erichson (Nitidulidae)	09/2007/455	1	Fiji	2007	adult	Intercepted	n/a	−	ns
*Carpophilus oculatus* Murray (Nitidulidae)	09/2009/3046	1	Tonga	2009	adult	Intercepted	n/a	−	ns
*Carpophilus* sp. (Nitidulidae)	T16_204A	1	New Zealand	2016	adult	*Citrus* sp.	n/a	−	ns
*Carpophilus* sp. (Nitidulidae)	T16_207A	1	New Zealand	2016	adult	*Citrus* sp.	n/a	−	ns
*Dactylosternum abdominale* (Fabricius) (Hydrophilidae)	VAITC5375	1	Australia	2015	adult	Beehive	n/a	−	ns
*Dermestes ater* De Geer (Dermestidae)	VAITC5376	1	Australia	2015	adult	Beehive	n/a	−	ns
*Epuraea signata* Broun (Nitidulidae)	T16_00204B/207B	2	New Zealand	2016	adult	*Citrus* sp.	82–83	−	ANZN009-17 to ANZN010-17
	VAITC5428	1	Australia	2015	adult	Beehive	82–83	−	ANZN024-17
*Epuraea* sp. (Nitidulidae)	VAITC5429	1	Australia		adult	Beehive	82–83	−	ANZN025-17
*Epuraea* sp. (Nitidulidae)	T14_03072	2		2014	adult	Beehive	83–84	−	ANZN004-17 to ANZN005-17
*Urophorus humeralis* (Fabricius) (Nitidulidae)	09/2009/27032	1	Tonga	2009	adult	Intercepted	n/a	−	ns
	T16_207C	1	New Zealand	2016	adult	*Citrus* sp.	84	−	ANZN011-17
	VAITC5373–5374	2	Australia	2015	adult	Beehive	84	−	ANZN019-17 to ANZN020-17
*Apis mellifera ligustica* Spinola (Apidae)	T15_06598	2	New Zealand	2015	adult	Beehive	n/a	−	ns
*Varroa destructor* Anderson & Trueman (Varroidae)	AV1/AV2	2	New Zealand	2016	adult	Beehive	67–68	−	MG793455
*Acarapis woodi* (Rennie) (Tarsonemidae)	AQ1	1	Canada	2015	adult	Beehive	67–68	−	MG793456
*Acarapis externus* Morgenthaler (Tarsonemidae)	Ae52/Ae126	2	New Zealand	2016	adult	Beehive	67–68	−	MG793457
*Acarapis dorsalis* Morgenthaler (Tarsonemidae)	AQ7	1	New Zealand	2015	adult	Beehive	68–69	−	MG793458

Note:

^*^larvae were identified to family morphologically, and to genus or species molecularly.

^**^VAITC: Victorian Agricultural Insect Tissue Collection, pinned specimen in VAIC;

^***^The sequences identities were calculated based on the 5′ fragment of the COI gene of the SHB samples from Australia.

nt: not tested. + : positive in the real-time PCR; −: negative in the real-time PCR assay. ns: no sequences obtained.

Those beetle samples not collected from Beehive were collected in New Zealand for testing the specificity of the real-time PCR assay.

### Phylogenetic relationship and morphological comparison of SHB from different locations

**Figure 2:**
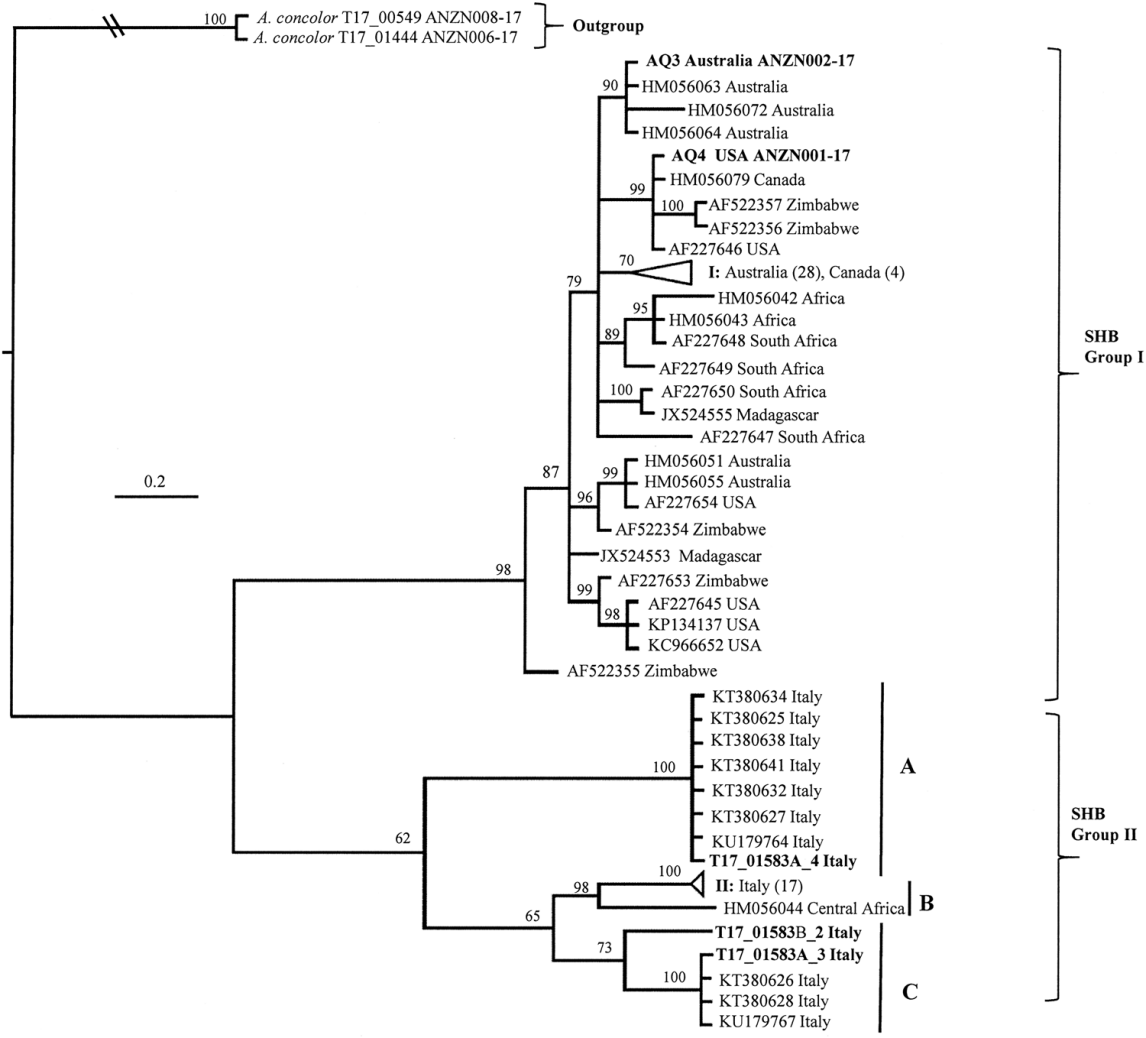
Bayesian phylogenetic analysis of 3′ fragment of COI sequences from 89 SHB using *A. concolor* as outgroup, with posterior probabilities of branches at each node. Bold letters for the taxa indicate sequences obtained in the current study (further details in Table 2). The accession number for each sequence is listed in the tree, except that the clades with similar sequences were collapsed for clear view of the tree. Collapsed clade details, I: Australia (28) - HM056045-50, HM056052-54, HM056056-62, HM056065-71, HM056073-74, AF227651-52, JX524554, Canada (4) - HM056075-78; II: Italy (17) - KU179765, KU179666, KT380635-KT380637, KT380633, KT380624, KT380629-KT380631, KT380639, KT380640, T17_01583A_1, T17_01583A_2, T17_01583B_1, T17_01583B_3 and T17_01583B_4. The numbers in bracket are the numbers of sequence in the clade.

**Figure 3:**
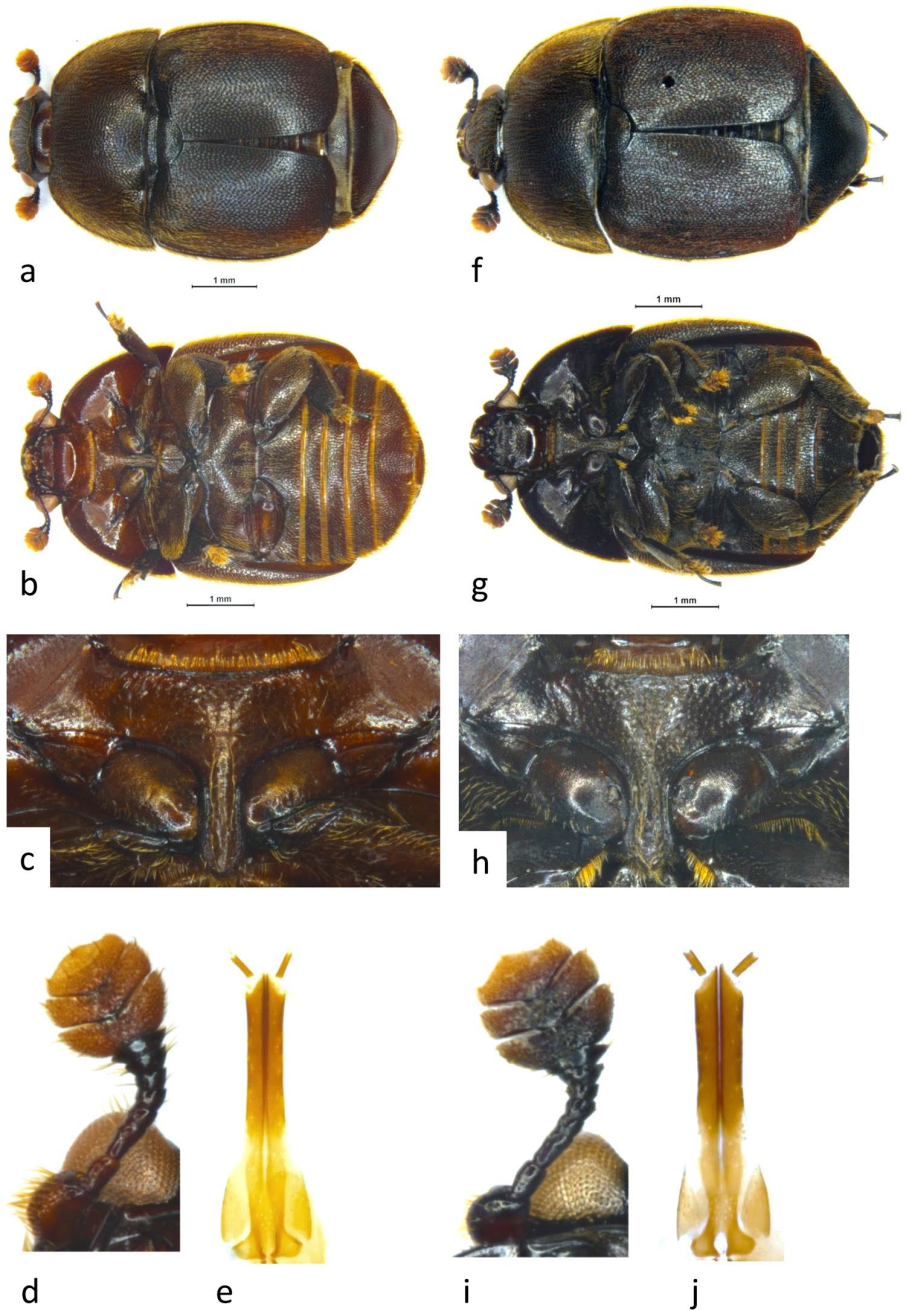
Small Hive Beetle from Italy (**a**) dorsal habitus, (**b**) ventral habitus, (**c**) prosternum, (**d**) ventral antenna, (**e**) ventral ovipositor; Small Hive Beetle from Victoria, Australia (**f**) dorsal habitus, (**g**) ventral habitus, (**h**) prosternum, (**i**) ventral antenna, (**j**) ovipositor. The images were taken by one of the co-authors.

Nearly full COI gene sequences for the SHB intercepted in New Zealand (AQ3 and AQ4), obtained from Italy (T17_01853A and T17_01853B) and *A*. *concolor* collected from Auckland were also obtained (Table 2). Phylogenetic analysis of AQ3, AQ4, T17_01853A, T17_01853B and the 79 SHB (Accession numbers shown on Fig. 2) downloaded from GenBank was constructed by using *A*. *concolor* as outgroup. Due to the short overlap of the sequences, the specimens with only 5′COI sequences obtained in this study were not included (Fig. 2). The phylogenetic tree showed that SHB formed two clusters (Fig. 2, Group I and II), the SHB sequences obtained from Australia, USA and South Africa formed a separate clade (Group I) from the recent detection of Italian SHB sequences (Group II). The one sequence (HM056044) of SHB from Cameroon, Central Africa also belonged to the same clade as the Italian samples (Group II). Between the two clades, there are around 4–6% differences in the COI sequences (~900 bp). Despite the genetic differences outlined here between SHB Groups I and II (Fig. 2), the recent Italian SHB was morphologically identified as *A*. *tumida*^11^ and no obvious morphological differences were observed (Fig. 3).

Following further examination in this study of SHB specimens from Italy and Australia, most characteristics were found to match with the description of *A*. *tumida* as described in Lee *et al*.^22^. Minor morphological differences were observed between the SHB specimens examined from Italy and Victoria (Australia), including difference in body colouration. Body colour was reddish/dark brown (particularly ventrally) in the Italian specimens (Fig. 3a,b) but black/dark brown in the Australian specimens (Fig. 3f,g). The shape of the prosternal process also differed between specimens, with lateral margins appearing parallel sided and more slender in the Italian specimens (Fig. 3c) but slightly more concave laterally in some Victorian (Australian) specimens (Fig. 3h). Finally, the ovipositor was narrower in the Italian specimens (Fig. 3e) compared with the Australian specimens (Fig. 3j). Some of the differences are subtle and may represent natural morphological variation, or be consequences of the size or life stage of individuals (eg. teneral specimens are often paler in colour) or be due to preservation conditions.

### Real-time PCR design

A total of 2, 329 COI sequences of SHB and closely related Nitidulidae species were downloaded from the GenBank and BOLD databases, the alignment showed that over 1,000 sequences included mainly the 3′ fragment of COI gene region. Amongst the sequences, 79 sequences were from SHB (3′ COI region), thus this region was used for real-time PCR assay design. The COI sequences of SHB were highly AT rich, with ~70% AT content. Due to the high AT richness, a BHQplus probe was designed, in which propyne modified bases (pdU and pdC) were incorporated and higher Tm was obtained using shorter bases^50^. The primers/probe have high matches to all the SHB COI sequence online and those we obtained in this study. The details about the real-time PCR primers/probe were: forward primer (Atum-3F: 5′-CCCATTTCCATTATGTWYTATCTATAGG-3′), reverse primer (Atum-3R: 5′-CTATTTAAAGTYAATCCTGTAATTAATGG-3′) and probe (Atum-3P: 5′-pdTApdTpdTpdTGCpdTApdTpdTApdTAGpdCpdCGGApdTpdTpdTGpdT-[BHQplus]-3′). The position in HM056074 are 635–662 for forward primer; 668–692 for probe and 731–703 for reverse primer. The PCR product was 97 bp, including primer binding regions.

### Real-time PCR optimization

The real-time PCR assay was initially tested with different annealing/extension temperatures using DNA extracted from the Australian (adult) and the USA (larva) specimens. The *Cq* of ~24 and ~17 cycles for the two DNA extracts were obtained, respectively from the temperatures of 56° to 60 °C. The *Cq* values of around 25 and 19 cycles for the temperature at 62.4 °C while around 33 and 26 cycles for temperature at 64.3 °C, no amplification was observed at the temperature of 66 °C. As a result, the optimal annealing/extension temperature was chosen as 60 °C. The PCR cycling conditions are 95 °C for 2 min, followed by 40 cycles of 95 °C for 15 sec, 60 °C for 60 sec.

The real-time assay was demonstrated to perform consistently well using different PCR mastermixes, with similar *Cq* values of around 17–18 cycles obtained for the DNA extracted from the USA specimen although slight differences in intensity were observed with the addition of MgCl_2_. There was negligible difference in the *Cq* values (~17–18 cycles for the USA specimen) when the assay was run with different concentrations of primers (125–300 nM) or probe (125–250 nM). Therefore the real-time PCR condition for SHB were chosen as: primer and probe concentration of 250 nM and 200 nM, respectively.

### Real-time PCR sensitivities

**Figure 4:**
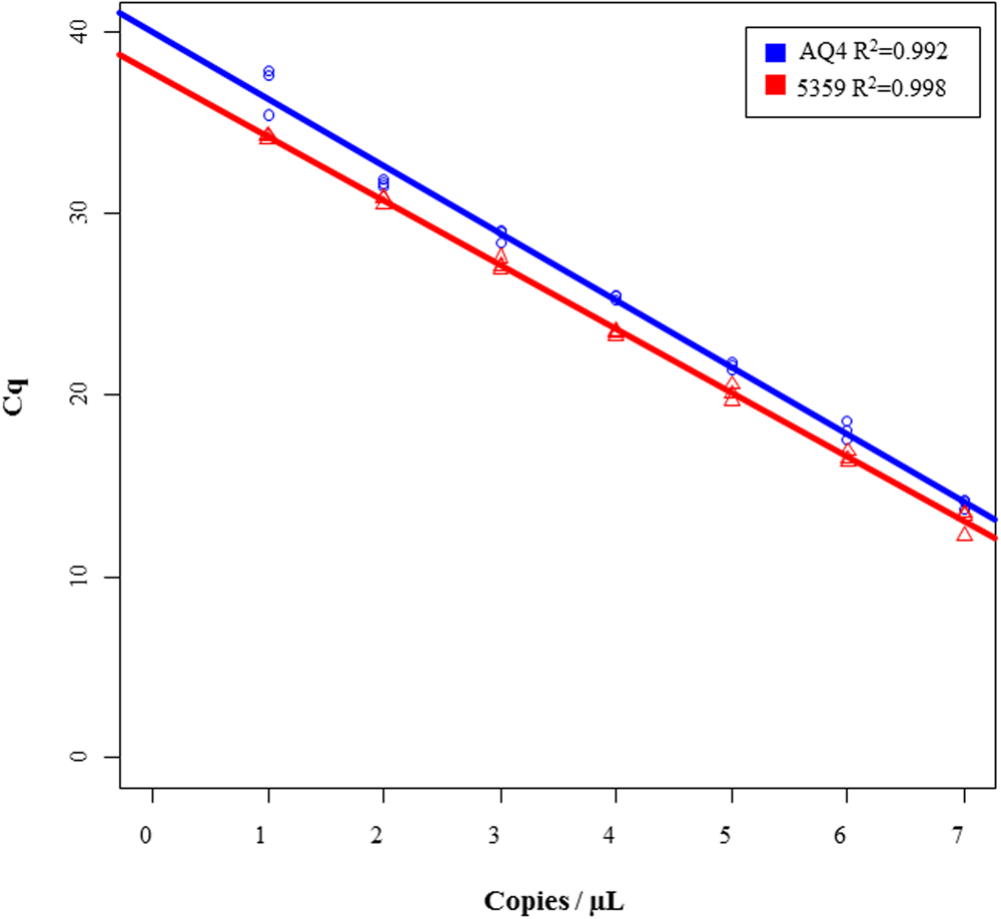
Sensitivity analysis of real-time PCR assay for the identification of small hive beetle using plasmid DNA containing COI inserts. Plasmid containing COI insert of SHB were series diluted to create calibration curves for sensitivity calculations. The standard curve built from *Cq* values against the log copy number (range = 10^7^–10^0^ copies) of COI insert (n = 3). The 95% confidence intervals of the slopes were plotted with a blue line for VAITC5359 (Australia sample) and a red line for AQ4 (USA sample). The *R*^2^ = 0.993 for VAITC5359 and 0.998 for AQ4 were obtained for the assay.

The linear dynamic range for the assay was tested on plasmids containing the COI inserts and extended from 10^7^–10^0^ copies of plasmid DNA. The 95% confidence limits of the linear dynamic range were plotted in Fig. 4, with a strong correlation coefficients (r^2^ = 0.993 and 0.998). The limit of detection (LOD) for the assay was estimated to be 100 copies/µL of target DNA. A template concentration of 10 copies/µL was sporadically detected in the assay with an average *Cq* value around 35 cycles. The calibration curves shown in Fig. 4 was able to detect 100% of the samples (and replicates) at the 100 copies/µL. This assay could also reliably detect up to 1:10^4^ and 1:10^5^ dilution of the DNA extracted from an adult leg (VAITC5360) and a small pieces of larva (AQ4) of the SHB (data not shown) specimen, respectively.

### Real-time PCR specificity and robustness

All small hive beetle samples were successfully amplified by the real-time PCR assay, while no amplifications were observed for the non-target species (Table 2). The assay was able to accurately identify small hive beetle samples from Australia, Italy and USA. No cross-reaction was observed with the DNA extracted from honey bee samples, varroa mites (*Varroa destructor*), and *Acarapis* mites (*Acarapis woodi, A*. *dorsalis* and *A*. *externus*).

All sixteen samples tested in the blind panel test were correctly identified as either positive or negative for SHB (Table 3). All the SHB samples were tested positive with *Cq* values less than 30. No amplification was observed in all the non-target species (Table 3).

**Table 3:** Blind panel tests for the real-time PCR assay against *Aethina tumida*.

Sample^#^	Species	Family	Year	Origins	PCR results (*Cq*)
BA1	*Carpophilus* sp.	Nitidulidae	2016	Auckland, NZ	0
BA2	*Aethina tumida*	Nitidulidae	2015	VIC, Australia	21.2
BA3	*Epuraea signata*	Nitidulidae	2016	Auckland, NZ	0
BA4	*Urophorus humeralis*	Nitidulidae	2015	VIC, Australia	0
BA5	*Aethina tumida*	Nitidulidae	2015	VIC, Australia	23.6
BA6	*Carpophilus* sp.	Nitidulidae	2016	Auckland, NZ	0
BA7	*Acarapis externus*	Tarsonemidae	2014	NZ	0
BA8	*Epuraea signata*	Nitidulidae	2016	Auckland, NZ	0
BA9	*Dactylosternum abdominale*	Hydrophilidae	2015	VIC, Australia	0
BA10	*Brachypeplus* sp.	Nitidulidae	2015	VIC, Australia	0
BA11	*Carpophilus* sp.	Nitidulidae	2016	Auckland, NZ	0
BA12	*Apis cerana*	Apidae	2015	NZ	0
BA13	*Aethina tumida*	Nitidulidae	2014	USA	18.3
BA14	*Apis mellifera mellifera/ligustica*	Apidae	2015	NZ	0
BA15	*Apis cerana cerana*	Apidae	2013	China	0
BA16	*Aethina tumida*	Nitidulidae	2014	Australia	25.4

Note: *Cq*: The cycle at which the fluorescence of a sample first cross the threshold line (ie exceeds background level). It is a measure for a positive reaction in real-time PCR reaction.

### Comparison with the SHB-specific PCR assay

DNA samples which tested positive in the real-time PCR assay developed in this study were used as templates in the SHB-specific PCR described in Idrissou *et al*.^51^. The expected size of ~193 bp PCR products were detected in the DNA extractions from Australia and the USA specimens, but very weak amplification was observed in the DNA samples from Italian specimens under the same PCR compositions and cycling conditions with and without additional MgSO_4_ added. Further tests showed that detectable PCR bands were observed in all the samples tested when the PCR cycles increased to 40 or the annealing temperatures reduced to 52 °C (data not shown).

The sensitivity of the SHB-specific PCR^51^ was tested against DNA extracted from the Australian (VAITC5360, adult) and the USA (AQ4, larva) specimens and compared to that of our real-time PCR assay. The SHB-specific PCR successful amplified DNA samples up to 1:10^2^ and 1:10^3^ dilutions whereas our real-time PCR assay successfully amplified the DNA in samples with up to 1:10^4^ and 1:10^5^ dilutions (see section on Real-time PCR sensitivities) from the Australian and the USA specimens, respectively. Therefore the real-time PCR developed in this study demonstrated over 100 times the sensitivity of the PCR assay by Idrissou *et al*.^51^.

## Discussion

This study generated the first DNA barcoding sequences (5′ COI) of SHB (Table 2), which will be useful not only for SHB identification but also for the future phylogenetic studies of other *Aethina* species with the sequences in the same region. DNA barcoding of the 5′ fragment of COI region of the SHB sample from Australia and USA were highly similar with more than 99% identity (Table 2), but they are different from sequences of the Italy SHB samples (Table 2) by around 4–6% in the Folmer’s region.

Phylogenetic analyses of DNA sequences of the 3′ fragment of the COI gene (~900 bp) revealed divergences among SHB (Fig. 2). COI sequences were highly similar for the SHB detected in Australia, Canada, Madagascar, South Africa, USA and Zimbabwe^20,47^, but different from those detected in Italy and one from Cameroon, Central Africa, HM056044 (Fig. 2 Group II). Similarly, Lounsberry *et al*.^20^ pointed out that the SHB COI sequence (HM056044) was 4.6% divergent from the rest samples they analysed. Our study demonstrated that this single Central African COI sequences is similar to the recent detection of SHB in Italy and formed in one clade (Fig. 2). In the Italy SHB samples, there are mainly three sub-clades formed, the central African sequence (HM056044) was close to the subclade B and C than those of sub-clade A. The COI sequences (~900 bp) for the sequences in the sub-clades B and C are less than 2% difference while they are different from the sub-clade A by around 3.3%. Therefore it suggested that the Italy detection is more related to the SHB from Cameroon, not the SHB from the other locations studied^20,47^. This is a novel finding that two genetically divergent groups are present within SHB populations. Questions surrounding the divergence of these two groups requires further investigation. An intensive morphological study of SHB from Italy, Australia and from around the world, including the original African source populations, would help to better define morphological variability within this species or, given the large sequence difference observed between the two SHB groups (Fig. 2), potentially reveal two closely related species. However, to date the two groups have been identified morphologically as SHB and both appear to cause serious damage to beehives^11^.

For a reliable assay, the primers/probe need to match COI variation from all the SHB specimens, but possess sufficient mismatches to the closely related species. Our assay was designed by aligning all the COI sequences from Nitidulidae, the sequences analysis showed that the majorities of COI sequences for SHB were toward to the 3′ fragment of the COI gene. To be able to capture all *A*. *tumida* sequences, our real-time PCR protocol was designed by targeting 3′ fragment of the COI gene (including SHB Groups I and II, Fig. 2). The primers were designed with degeneracy in order to capture all the haplotypes of the *A*. *tumida* sequences. *In silico* tests showed that the primers (Atum_3F and Atum_3R) and probe (Atum_3P) have 5, 5 and 2 mismatches with *A*. *concolor*, there was no cross-reaction observed in the real-time PCR assay either (Table 2).

**Figure 5:**
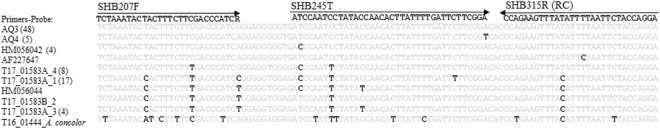
Sequence alignment of the primer binding sites for the primers/probe for SHB sequences developed by Ward *et al*.^48^ compared with current known SHB haplotype variations. The primers and probe names for the real-time PCR assay from Ward *et al*.^48^ are listed in the top of the sequences. The reverse complement sequences for reverse primer SHB315R (TCCTGGTAGAATTAAAATATAAACTTCTGG) is listed as SHB315R(RC). All the SHB sequences were compared and the regions for the primers/probe were extracted and analysed. This figure only showed the unique sequences for this region and one of the accession number is listed. The numbers of the taxa with the identical sequences in the primers/probe binding region are listed in the bracket. The mismatches of the primers and probe with the SHB sequences are showed in bold letters. *A concolor* sequences were also listed for comparison.

A real-time PCR protocol was developed in 2007, in which the probe, SHB245T is 39 bp in length^48^. The design for the real-time PCR was more likely due to the high AT rich sequences, thus longer probe was needed to be provide a higher Tm. As probe length increases, the distance between the fluorophore reporter and quencher at the probe end increases, resulting in reduced quenching and thus, poor signal to background discrimination will be observed. Therefore, the probe longer than 30 bases are unlikely to have efficient quenching as an end-labelled probe. Longer probes are also more likely to bind non-specifically, and therefore exhibit reduced specificity. Prior to develop our real-time PCR assay, attempts were made to synthesise the primers/probe from Ward *et al*. ^48^, but most companies suggested that an internally quenched probe is needed for probe over 30 bases. In addition, there were only 15 COI sequences available when this assay was designed^48^, thus it was not necessary to order the primers/probe by Ward *et al*.^48^ and validate the real-time PCR assay. On this other hand, the real-time PCR assay designed in this study based on 79 SHB sequences, containing new divergent haplotypes of SHB sequences (Fig. 2). In *in silico* analysis, mismatches in the binding sites for primers and probe in Ward *et al*.^48^ were also observed in 31 (23 from GenBank and 8 from this study) sequences (Fig. 5). Therefore it was necessary to develop a new real-time PCR assay for the rapid detection of SHB.

The real-time PCR assay developed in this study covers the known COI diversity (i.e. including Groups I and II, Fig. 2) of haplotypes of SHB available (89 sequences) using the BHQplus probe technique (http://eu.biosearchtech.com/products/bhqplus-probes). The incorporation propyne modified bases of T and C^50^ could achieve a higher Tm with a shorted sequence in the AT rich *A*. *tumida* COI sequence. Using this technique, a BHQplus probe of 25 bases was able to reach a sufficiently high Tm and allow the assay to effectively amplify the SHB samples and increase its sensitivity. The real-time PCR assay has also demonstrated its sensitivity by detecting the SHB samples in less than 30 cycles of *Cq* values detested in this study.

Comparison with the recent published PCR assay developed by Idrissou *et al*.^51^ indicated that the real-time PCR assay developed in this study is more robustness in amplifying different haplotypes of SHB samples. When testing with the SHB-specific PCR assay, successful amplification using the Australian and the USA specimens, but very weak and not obvious amplifications were observed using the DNA extracted from the Italy specimens. Further *in silico* testing showed that the two primers (AT420F and AT623R) designed by Idrissou *et al*.^51^ were based on one sequence obtained from *A*. *tumida* isolate BRL-Maryland unplaced whole genome genomic scaffold (NW_017855158.1), which represents one of the COI haplotypes. As there are several different haplotypes detected in the SHB specimens (Fig. 2), there are mismatches observed in the two primers: 2–3 mismatches in the AT630R primer for the available SHB COI sequence (SHB Group I and II in Fig. 2); for the AT420F, however, there are 2 mismatches in the 5′-end for the Group I while 1 in the 5′-end, 1–2 in the 3′-end mismatches for the Group II SHB specimens (Fig. 2). Therefore the SHB-specific assay^51^ might provide a false negative results, for example, no detectable PCR products were observed in the PCR reactions using the DNA from some of the Italy specimens tested in this study under the condition suggested in Idrissou *et al*.^51^. The reasons might due to (1) the mismatches in the 3′-end (critical position) of the AT430F for the Italy SHB reduced the amplification efficiently; (2) different PCR mastermixes were used in the reaction, affecting the assay robustness. Obvious detectable PCR products were observed when increasing the PCR cycles or reducing the annealing temperatures further indicated that the amplification efficiency are low. If this real-time PCR will be applied for diagnostic, further validation are needed.

In comparison to the SHB-specific PCR assay, the real-time assay demonstrated its sensitive by detecting 100 times less concentrated DNA samples than the PCR assay^51^. Furthermore, it is a closed-tube system which no post-PCR manipulations was required, thus reducing the risk of cross contamination between samples. Therefore the real-time PCR assay developed in this study will enable the quick, reliable and highly sensitive detection of SHB smaples.

Application of the real-time assay in the New Zealand quarantine framework provides an alternative to morphological identification, especially useful when immature stages of the beetles and the damaged specimens are intercepted. The real-time PCR assay targeting SHB developed in this study is conformant with the MIQE guidelines for qualitative assays^49^. High specificity and sensitivity are much desired in border diagnostics, the assay showed high efficiency and sensitivity in detecting the target species. In the specificity tests, all the SHB samples from Australia, Italy and USA were correctly identified, no cross-reactions with the closely related species were observed. The assay has tested on *A*. *concolor* from New Zealand, but no other *Aethina* species were available to validate the real-time PCR assay. However, *in silico* analysis of the *Aethnia* species with the COI sequences showed that SHB is over 17% different compared with *A*. *suturalis* (KJ480791). Therefore, the possibility of cross-reaction with *A*. *suturalis* are extremely low. The beetle samples associated with bee hives were also tested in the real-time PCR assay, no amplification observed (Table 2), further indicating the specificity of the real-time PCR assay. This real-time PCR assay could assist in rapid identification of SHB, including the immature stages of SHB which are difficult to be identified by morphological features^52^.

The real-time PCR assay developed here could detect SHB from Australia, Italy and USA of different life stages, no cross reactions were observed with honeybees, bee mites (*Acarapis woodi, A*. *externus* and *A*. *dorsalis*) and varroa mites. The assay was also tested against beetles of genera *Brachypeplus*, *Carpophilus, Dactylosternum, Dermestes, Epuraea* and *Urophorus* (Table 2), and no-cross reactions were observed. This assay tested against the nitidulid beetle samples intercepted and related species detected in beehives at New Zealand borders and post border, no amplification was observed, therefore it could be applied to the routine diagnostic for SHB at the New Zealand borders.

There are several exotic beetle species which are associated with bee hives (Table 1) that have not been tested in our real-time PCR assay due to the difficulties in obtaining those specimens. However, *in silico* analysis showed that these species possess a divergent COI region to that of the SHB. For example, there are more than 15% differences between SHB and the species of *Carpophilus lugubris, Cychramus luteus* and *Glischrochilus fasciatus* when comparing their COI sequences. *Cryptophagus hexagonalis* (Coloptera: Crytophagidae), the only one not in the family Nitidulidae (Table 1), was reported in the beehive^53^, no DNA sequences available for this species, but there are sequences for this genus. Sequence comparison of SHB with *Cryptophagus* spp. showed that the similarities with SHB are 77–80%, thus indicating *Cryptophagus* spp. are more distant from SHB. Therefore we are confident that this real-time PCR assay for SHB is not likely to be able to amplify those beetle species. In addition, no other *Aethina* species could be obtained for testing the real-time PCR assay besides *A*. *concolor*. There are limited studies on the genus, DNA barcoding reference sequences (on BOLD) are currently available only for *A. timuida*, *A. concolor* and *A. suturalis*. The real-time PCR assay was designed based on the current available sequences, tested and validated with the species we could obtain, therefore further tests and analysis will be conducted if more related species obtained and DNA sequences available.

Here, we report the DNA barcoding identification of SHB, *A*. *tumida* and other related beetle specimens, including *A*. *concolor*, *Brachypeplus* sp.*, Epuraea signata, Epuraea* sp. and *Urophorus humeralis* (BOLD project ANZN), which enrich the reference sequences for the nitidulid beetles in BOLD and GenBank databases. The real-time PCR assay developed for SHB in this study is suitable for routine application by diagnostic and research agencies, for facilitating exports and imports. It will assist in border security agencies worldwide to limit and monitor the spread of this pest. This assay provides a rapid, accurate and specific alternative to morphology, or more time intensive DNA barcoding methods, for identification for SHB. This assay has been fully optimised for immediate deployment in New Zealand and Australia, and should prove useful for SHB surveillance in other regions of the world.

## Material and Methods

### Sampling

Small hive beetle samples used in this study were collected from sticky mats used for bee hive pest surveillance in Australia, intercepted at the New Zealand borders and obtained from Italy (Table 2). Additional nitidulid beetles for DNA extraction include pinned specimens in the Victorian Agricultural Insect Collection (VAIC, Melbourne, Australia), frozen and ethanol (75% and 96%) preserved specimens from the Plant Health and Environment Laboratory collection (PHEL, Auckland, New Zealand) (Table 2). All the specimens were initially identified morphologically and some were also confirmed through DNA barcoding, using COI sequences (see section 4.2 below).

Four adult specimens of SHB collected from Italy (ANZN026-17 to ANZN029-17) and four specimens from Victoria, Australia (VAIC 63240, 63244, 74457 and 78224) were closely examined and compared to the morphological description and keys in Lee *et al*.^22^. Three females (one Italian and two Australian) were dissected for examination of the genitalia. Specimens were first soaked in 10% Potassium Hydroxide (KOH) overnight before removing the ovipositor from the abdomen using fine micro-tools.

### DNA extraction, PCR amplification and sequencing

Total DNA was extracted using the DNeasy Blood and Tissue kit (Qiagen, Valencia, CA, USA) as per the manufacturer’s instructions. A single leg of adult or segment of larva was used for each extraction and physical disruption was performed by micro-pestles, with the final DNA eluted in 100 µL of AE buffer (Qiagen, Valencia, CA, USA). For DNA barcoding of the beetle species, LCO1490 and HCO2198 primers^46^ were used to amplify the PCR products of 5′fragment of COI gene from most specimens. The amplification of *A*. *concolor* and some of the specimens for 5′ fragment of COI gene using LCO1490 and HCO2198 was not successful, thus several pairs of primers were tested, including mtD-7.2 F/mtD-9.2R^54^, CI-J-1751/CI-N-2191 and CI J-1718F/COIREVA^55^. Additionally, older pinned VAIC specimens were amplified using two new “mini-barcode” internal primers, NitidulidaeCOI-F 5′-AAAGAGGAGCWGGMACWGG-3′ and NitidulidaeCOI-R 5′-GCGATATTGGATGAVAGDGG-3′. These primers were designed using Primer 3^56^ from COI sequences of *Carpophilus davidsoni* Dobson*, Urophorous humeralis*, Fabricius*, Brachypeplus* sp., *Aethina concolor* (Macleay, 1872), obtained from Brown *et al*.^57^, for amplification of the 5′ COI region in two approximately 350–400 bp (55 bp overlapping) amplicons (Fig. 1). To obtain the 3′ fragment of the COI gene sequences, selected samples were used for PCR with primers: A1859 and AT3014^58^ (Fig. 1).

For most PCR reactions, each 20 µL reaction consisted of 1× GoTaq master mix (Promega, Madison, WI), 250 nM of each primer, 0.5 µg/µL Bovine Serum Albumin (BSA, Sigma-Aldrich Co.), 2 µL of DNA extract. Cycling conditions were: initial denaturation at 94 °C for 2 min, 40 cycles of 94 °C for 15 sec, 50 °C for 30 sec and 72 °C for 60 sec, followed by final extension step of 7 min at 72 °C. PCR reactions employing NitidulidaeCOI-F/R in combination with LCO/HCO (Fig. 1) used 2 µL of template DNA in 25 µl PCR reactions, including: 1 × BSA, 1 × NEB ThermoPol Reaction Buffer, 2 mM MgCl2, 0.2 mM dNTPs, 0.5 μM of each primer and 1 unit of NEB Taq DNA Polymerase, with a PCR profile of 94 °C for 2 min, followed by 40 cycles of 45 sec steps at 94, 51 and 72 °C and a final extension step of 72 °C for 2 min. The amplicons were electrophoresed on 1.2% agarose (in 1 × TAE buffer) gel stained with SYBR^®^ safe (Life Technologies™), and visualised using a Gel Doc Software system (BioRad, Hercules, CA, USA). Amplified products were sequenced bi-directionally using the amplification primers for 5′ fragment (as above) and AT2380S and AT2519A^47^ for the 3′ fragment of COI gene by EcoGene^®^ (Auckland, New Zealand) or Macrogen (Seoul, South Korea). The obtained DNA sequences were edited and aligned using Geneious Pro 9.1.5 (http://www.geneious.com, Kearse *et al*.^59^) and BLAST searched against the GenBank database^60^ or BOLD database^45^ to confirm morphological identifications.

The COI sequence data obtained in this study were deposited in BOLD and GenBank databases (Table 2). BOLD accessions include full collection details along with diagnostic images of VAIC specimens obtained using a Leica M205C microscope and DFC450 camera, those of PHEL photographed using a Zeiss AxioCam HRc camera attached to a Zeiss interference-phase contrast microscope (Axio Imager 1), and can be found in the BOLD project Australian and New Zealand Nitidulidae - ANZN.

### Phylogenetic relationship of SHB samples

Sequence data of COI gene from 79 SHB samples were downloaded from the GenBank database. Ten nearly full length (AQ3, AQ4, T17_01583A_1 to T17_01583A_4 & T17_01583B_1 to T17_01583B_4) and nine SHB 5′ fragment of COI sequences were obtained in this study (Table 2). Multiple sequence alignment was performed using the Geneious aligner and re-aligned with Clustal W in Geneious version 9.1.5 (http://www.geneious.com, Kearse *et al*.^59^). The alignment of SHB samples (89 samples) with mainly 3′ fragment of COI gene were used, but due to the small region of sequence overlap, some of the Australian samples with only the 5′ fragment of sequences were not used in phylogenetic analyses. Phylogenetic trees were constructed using Neighbour-Joining (NJ), Maximum-Likelihood (PHYML) and Bayesian (MrBayes) methods in Geneious Pro 9.1.5 under the default settings^61^. Multiple runs were performed using the model GTR and rate variation invgamma. For Bayesian tree estimation, the resulting perimeter files were inspected for chain convergence and mixing in Tracer 1.4^62^. The trees were rooted using *A*. *concolor* COI sequences (T6_01444 & T17_00549) obtained in this study as outgroup.

### Real-time assay design

All currently available COI sequences of Nitidulidae, including *A*. *tumida* and closely related species were downloaded from GenBank and BOLD databases (up to June 2016). The sequences were aligned in Geneious version 9.1.5 (http://www.geneious.com, Kearse *et al*.^59^). Suitable regions for probe and primer design were identified manually. The probe was designed using Oligo Evaluator from Biosearch (https://rtd.biosearchtech.com/design/OligoEvaluator.aspx), and primers were designed manually. Melting temperature (Tm) and secondary structures of primers and probe sequences were evaluated using OligoAnalyzer 3.1 from IDT Integrated DNA technologies (https://sg.idtdna.com/calc/analyzer). The primers and probe were BLAST searched to test their specificity in GenBank and checked their matches to SHB sequences in Geneious 9.1.5 (http://www.geneious.com, Kearse *et al*.^59^).

### Development and validation of the real-time PCR assay

#### Optimization

The real-time PCR protocol was tested on a CFX96™ Touch Real-time platform (BioRad). Gradients of temperatures (56–66 °C), primer concentrations (200, 250 and 300 nM), probe gradients (125, 200 and 250 nM), with and without the additional MgCl_2_ (1.5 mM) were used to optimise the PCR conditions. To select an appropriate real-time mastermix for the assay, preliminary comparisons were trialled with commercially available mastermixes: PerfeCTa® qPCR ToughMix® (Quanta Bioscience), SsoAdvanced™ Probes Supermix (BioRad). The quality of each DNA sample was also tested with conventional PCR using COI (LCO1490 and HCO2198 primers)^46^ or 18S ribosomal RNA (rRNA) gene internal control real-time PCR (Applied Biosystems, CA, USA).

#### Sensitivity

To evaluate the analytical sensitivity of the developed assay, a 1,100 bp template COI PCR product, amplified as described in section 4.2 was used to prepare plasmid standards of known copy number. The amplicon was cloned using the TOPO^®^TA vector cloning kit (Invitrogen, Carlsbad, CA, USA) as per the manufacturer’s instructions. Cloning was performed from the PCR products amplified from DNA extracted of two small hive beetles, one from USA and the other one from Australia. The two clones containing the correct insert were selected for preparing standards. Plasmid DNA were extracted using the Wizard^®^ Plus SV Miniprep (Promega, Madison, WI, USA). The plasmid DNA was quantified using a µDrop plate in MultiSkan GO DNA quantification system (Thermo Scientific, USA) and normalised to a concentration of 10^7^ copies/µL. A dilution series of the plasmid from 10^7^–10^0^ copies was created using sterile TE buffer (Sigma). Analytical sensitivity of the assay was determined using the dilution series with each concentration in triplicate per reaction. Linear regression was performed between the quantitative cycle (*Cq*) and the log_10_ of the copy number, measuring the fit as *R*^2^. Amplification efficiencies for individual reactions were calculated using the formula, E = 10^|1/slope|^ and converted to E_%_ by (E − 1) × 100 in the R environment version 3.1.1^63^.

#### Specificity and robustness

Closely related (i.e. Nitidulidae) species and other species collected from bee hives were used for the specificity test of the assay (Table 2). DNA samples extracted from honey bee (*Apis cerana cerana*), tracheal mites and varroa mites were also used to test whether there were cross-reactions. In addition, DNA extracted from other beetles were tested in the real-time PCR assay (Table 2), include Nitidulidae: *Brachypeplus* sp., *Carpophilus hemipterus* (Linnaeus), *Carpophilus oculatus* Murray, *Epuraea signata* Broun, *Epuraea* sp., *Urophorus humeralis* (Fabricius); Dermestidae: *Dermestes ater* De Geer and Hydrophilidae: *Dactylosternum abdominale* (Fabricius).

Sixteen samples were prepared for the blind panel test (Table 3), with the operator having no knowledge of the sample identities. The samples were tested in the real-time PCR protocol for small hive beetle, each samples were tested in duplicate, including the positive and water controls.

### Comparison with the SHB-specific PCR assay

The reliability and sensitivity of the real-time PCR assay developed in this study was compared with the published SHB-specific conventional PCR assay by Idrissou *et al*.^51^. The PCR primer set, AT420F and AT623R were ordered and used for PCR with the DNA extraction from SHB of Australia, the USA and Italy, under the same PCR compositions and cycling conditions as per Idrissou *et al*.^51^ using GoTaq master mix (Promega, Madison, WI) with and without additional 1.5 mM MgSO_4_. Further testing using the PCR compositions with additional 1.5 mM MgSO_4_ were conducted under two PCR cycling conditions, (1) PCR cycles changed to 40 instead of 35 cycles, and (2) the annealing temperature decreased to 52 °C instead of 56 °C for 35 cycles. The resulted PCR products were gel electrophoresis with 2% agrose in 1 × TAE buffer, The PCR sensitivity was also compared with series dilutions of DNA extraction from SHB samples.
